# Biomarkers in inflammometry pediatric asthma: utility in daily clinical practice

**DOI:** 10.1080/20018525.2017.1356160

**Published:** 2017-08-09

**Authors:** Silvia Sánchez-García, Alicia Habernau Mena, Santiago Quirce

**Affiliations:** ^a^ Allergy Section, Hospital Infantil Universitario Niño Jesús and Health Research Institute La Princesa, Madrid, Spain; ^b^ Allergy Section, Hospital de Mérida, Badajoz, Spain; ^c^ Department of Allergy, Hospital Universitario La Paz and CIBER of Respiratory Diseases (CIBERES), Madrid, Spain

**Keywords:** Asthma, biomarkers, diagnosis, phenotype, severe asthma, nitric oxide, IgE, periostin, children, eosinophilia

## Abstract

Asthma is a common disease in both high and lower income countries that starts early and persists often for life. A correct and accurate diagnosis, treatment and follow-up during childhood are essential for a better understanding of adult asthma and avoiding over- or under-treatment.

Th2 inflammation in children with asthma symptoms is usually assessed by measuring with serum total IgE, blood eosinophilia and FeNO levels that may help to predict asthma, particularly in those infants and young children in whom lung function tests are difficult to perform. FeNO measurement, compared to intra-individual levels, may be useful also for ascertaining treatment adherence. Nevertheless, an isolated measurement may be insufficient and only the combination of these markers improves the diagnosis, phenotyping and follow-up of an asthmatic child.

## Introduction

Asthma is a common disease in both high and lower income countries that starts early and persists often for life.[1] A correct and accurate diagnosis, treatment and follow-up during childhood are essential for a better understanding of adult asthma and avoiding over- or under-treatment.

During recent years, a huge quantity of studies of biomarkers have been published, but with no clear conclusions.[2] Moreover, their clinical utility is not well clarified. It is broadly acknowledged that an ideal biomarker does not exist. However, in daily clinical practice, it would be desirable to have a universal, cheap and non-invasive test able to predict which infants will develop asthma in the future, to diagnose asthma, to monitor treatment adherence, to provide guidance during the follow-up and even to predict long-term evolution. This does not currently exist, as asthma is a complex entity with multiple physiopathology.

## Serum total IgE (tIgE)

Serum total IgE does not meet the criteria of an ideal biomarker. As an indicator of allergic asthma it is appropriate, reproducible and does not present hourly variability and can be measured easily and at low cost; however, its main restriction as biomarker is its low specificity for asthma, as it may be present in other allergic, parasitic and immune diseases. Similar to other biomarkers, tIgE may be an indicator of a biological (atopy) or pathological process (allergic disease).

### Utility as predictor of asthma development

Measurement of tIgE levels gives important information for the predisposition of atopic status, which has been additionally associated with asthma. More than 80% of children and adolescents with asthma exhibited an allergic component.[3–5] Also, asthma prevalence was higher in children whose parents presented increased levels of tIgE.[6,7]

Atopic infants have an earlier and higher rise of tIgE than age-matched non-atopic controls. The COAST study [8] clearly depicts the increased risk for later asthma development after bronchiolitis caused by rhinoviruses (RV) in infants with a family history of atopy. There is an increased FcεRI expression and lower RV-induced IFN responses in allergic asthmatics, when compared with non-allergic, non-asthmatic children.[9] IgE changes to the innate and adaptive immune response to the virus have been suggested as the most plausible reason.[10]

### Utility for asthma diagnosis

Several decades ago, elevated tIgE was linked to decreased pulmonary function.[11] More recently, it has been related to eosinophils and airway inflammation, being considered a relatively sensitive, but not specific, marker of lower airway eosinophilia and a poor marker of lower airway neutrophilia. Moreover, a positive correlation between tIgE levels and bronchoalveolar lavage (BAL) eosinophil counts has been found in asthmatic children.[12]

### Utility for asthma follow-up

In asthma management guidelines, the primary goal of treatment is asthma control and reduce future risk. To date, asthma control guided only by symptoms and lung function is not optimal in many children.[13] Carrol et al. [14] demonstrated a clear relationship between the stages of increased asthma severity and elevated levels of tIgE. They found significantly higher tIgE levels in asthmatic patients requiring hospitalization compared to non-hospitalized asthmatics and a significant relationship between FEV_1_ < 80% and higher IgE levels compared to patients with FEV_1_ > 80%. Other authors also associated tIgE levels with the severity of symptoms, the risk of exacerbations and the remodeling of the airway.[15,16]

Serum IgE is not a biomarker of response to therapeutic interventions. Although this value is needed in order to select candidates for omalizumab treatment in patients with moderate and severe allergic asthma, it is not useful as a predictor of response to the drug.

The recommendations in daily clinical practice are that isolated tIgE measurement is not useful for asthma diagnosis and/or follow-up. As a marker of atopic condition, it may be useful as a predictor of asthma development[17,18].

## Allergen-specific IgE (sIgE), skin prick test (SPT)

Atopy has been generally accepted as a risk factor for asthma onset, severity and worse prognosis in pediatric asthma.[17] Therefore, sensitization to allergens, resolved by skin prick test (SPT) or specific IgE (sIgE), is a marker for a long-term presence of allergy disease, such as atopic dermatitis, allergic rhinoconjunctivitis and/or asthma.[18]

### Utility as predictors of asthma development

Allergen-specific IgE (sIgE) and skin prick test (SPT) are both common markers for atopic disease.[19] Aeroallergen sensitization (measured either by SPT or sIgE) at the age of 2 may be a useful predictor for later allergy-related disease, like asthma, with a frequent onset at the age of 6 years.[18,20] Aeroallergen sensitization and egg sensitization during infancy has been demonstrated to be associated with an increased risk of asthma at 5 years of age.[21]

Likelihood of asthma is increased in patients sensitized to a wide number and diversity of allergens.[22] However, both total and sIgE show limited value, especially in tropical areas, where higher frequencies of sIgE were detected in helminth-infected children.[23] Moreover, geohelminth infections may modify the association between dust mite sensitization and wheezing in urban schoolchildren.[24]

Hammer et al. [25] recently studied several predictors of asthma control in a cohort of 209 children: sex, age, atopy, adherence to medication, educational issues and seasonal symptoms. Only atopy was identified as a significant predictor of uncontrolled asthma.

### Utility for asthma follow-up

Specific IgE levels and SPT aeroallergens can highlight asthma severity.[26] In a clinical setting, in children with severe, therapy-resistant asthma, both tests should be performed in order to detect sensitization.[27] The increase in number of allergens that patients are sensitized to may indicate also an increase in the severity of asthma.[28]

Dust mite sensitization has been reported in different populations as a marker of asthma severity, especially *Dermatophagoides pteronyssinus* and *Dermatophagoides farinae*.[29] Cockroach sensitization has been related to asthma morbidity, with a linear relationship with levels of sIgE. In fact, increased Bla g 1 in the home was a good predictor for sensitization; however this relationship was not demonstrated for Der f 1.[26]

Polysensitization to furry animals (mainly cat, dog and horse) was related to severe childhood asthma, and high sIgE was related to more severe disease, not only to whole extract but also with recombinants, particularly Can f 2 and Equ c 1.[30]

In summary, sensitization to allergens, particularly perennial aeroallergens such as house dust mites or furry animals, measured either by SPT or sIgE, are related to an increase in the severity of the disease.

### Recommendations in daily clinical practice

Sensitization to allergens measured both by SPT and sIgE are biomarkers of atopy, a condition that may predict future asthma in preschool children. Moreover, sensitization to certain perennial allergens (dust mites or animal dander) may be related to asthma severity.

## Blood eosinophilia

The value of eosinophil counts in blood has to be interpreted in relation to other biomarkers such as FeNO, reported symptoms, lung function and treatment.

### Utility as predictor of asthma development

Elevated blood eosinophils are robust predictors of asthma development.[18] The persistence of eosinophilia 4–6 weeks after respiratory syncytial virus infection predicts the increased risk of asthma in preschool and school life, according to a prospective study in 83 children after hospitalization for bronchitis.[31] Anderson et al. [20] analyzed non-invasive biomarkers to predict asthma in 244 children, and found that an eosinophil count ≥ 300 cells µl^–1^ and aeroallergen sensitization at age of 2 were associated with an increased risk of asthma when children were 6 years old. Blood eosinophilia (≥4%) is one of the criteria included in the Asthma Predictive Index and its modifications.[32] A recent systematic review has confirmed that blood eosinophilia is a significant risk factor predicting persistence of early wheezing in school aged children.[33]

### Utility for asthma diagnosis

Both blood eosinophils and eosinophil cationic protein (ECP) have been identified in patients with bronchiolitis, cystic fibrosis and asthmatic children.[34,35] Arron et al. [36] reported that using a threshold of 270 eosinophils^ ^µl^–1^ of blood, showed a positive and negative predictive value of 79% and 91%, respectively, for predicting sputum eosinophilia > 3% in adults with asthma. Blood eosinophils are not recommended as marker of airway eosinophils.[37]

### Utility for asthma follow-up

Busse et al. [38] related high eosinophil counts to good response to omalizumab in adults. According to a TENOR study,[39] comparing severely asthmatic children and adults, children had a higher frequency of visits to emergency departments, more sensitization to aeroallergens, elevated levels of peripheral blood eosinophilia and show higher tIgE levels.[40]

Asthmatic patients with significant eosinophilia are at higher risk of more severe disease.[41] Individuals with asthma (both children and adults) and blood eosinophil count greater than 300 cells µl^–1^ were more likely to report asthma attacks.[42]

The recommendations in daily clinical practice are that blood eosinophilia is useful as predictor of asthma development in children, monitoring response to inhaled corticosteroids and higher risk of severe disease.

## Fractional exhaled nitric oxide (FeNO)

During recent years, FeNO emerged as a promising biomarker for eosinophilic airway inflammation. Childhood asthma is often characterized by elevated FeNO. FeNO levels may be age-dependent, as an average increase of 7.4% per year of age has been reported, and they may also decrease with increasing body mass index (BMI).[43] According to the last GINA update,[44] FeNO may also oscillate during viral infections and decrease while bronchoconstriction is presented or during the early phase of an allergic response. This variability may complicate FeNO assessment by the clinician.

### Utility as predictor of asthma development

The last GINA guidelines update discloses that, among preschool children with recurrent coughing and wheezing, an elevated FeNO may predict physician diagnosis of asthma by school age.[44]

Children with asthma at 5 years of age had a greater increase in FeNO between infancy and 5 years compared with those without asthma. Measurement of FeNO early in life may provide important insights into the subsequent risk of asthma.[21] Bastain et al. prospectively followed for 3 years a large population of asthma-free children, finding out that FeNO was associated with increased risk of new-onset asthma. They also detected that children with the highest FeNO showed more increased risk of new-onset asthma compared to those with the lowest, this being more relevant if they had no family history of asthma.[45]

Elevated values of FeNO are associated with increased risk for asthma not only among the asthma-free population, but also in preschool children with lower airway symptoms.[46]

### Utility for asthma diagnosis

Sivan et al. [47] described a group of school-age children in whom FeNO was especially useful, finding that those with FeNO>19 ppb achieved a sensitivity of 80% and specificity of 92% for asthma diagnosis. Significant correlations were also identified between FeNO and FEV_1_ and between FeNO and FEF25-75.[48] Low FeNO levels in a pediatric population may help to rule out allergic asthma, whereas high levels may be due to allergic sensitization, older age, rhinitis, and lower BMI, in addition to asthma.[43] However, the main concern is that there is no consensus on the level of FeNO optimal to rule out asthma diagnosis.[49] Grzelewski et al. [50] suggested that FVC/FeNO, FEV_1_/FeNO, FEF25-75%/FeNO, FeNO/FVC, FeNO/FEV_1_, FeNO/FEF25-75% ratios show a better correlation with asthma diagnosis.

In summary, measurement of airway inflammation by means of FeNO can be useful and convenient for asthma diagnosis, particularly when bronchial challenges and/or spirometric maneuvers cannot be correctly performed.[49] Perzanowski et al. [51] have shown that indirect bronchial hyperresponsiveness (BHR) to adenosine 5ʹ-monophosphate reflects airway inflammation in terms of FeNO better than methacholine test. However, some authors observed that a single FeNO measurement is probably of scarce prognostic value and propose perform repeated measurements over time.[48]

There are limited number of publications dedicated to asthma in the adolescent population, as they are mostly inferred from adults or children studies. FeNO in asthmatics over 13 years old correlated significantly with only some of lung function parameters: MEF75, MEF50, MEF25 and PEF but not with FEV_1_ or FVC, which was not confirmed in school-age patients.[52,53] High levels of FeNO were also related to BHR in adolescents [54] and with an increased number of allergen sensitization.[55]

### Utility for asthma follow-up

Vijverger et al. [56] determined that high FeNO values were related to lower medication adherence rates, and, anecdotally, detected higher levels of FeNO among children living in a rural environment. FeNO measured after hospitalization due to an asthma exacerbation resulted useful in identifying children who will respond to inhaled corticosteroids therapy.[57]

Nevertheless, clinical trials monitoring inhaled corticosteroids treatment in the pediatric population have not benefited from adding FeNO to a symptom-based approach. This may be explained by a significant intrasubject change in FeNO values.[58]

The utility of FeNO as biomarker to monitor asthma control is still discussed today.[56] As has been debated previously, the attempt to characterize individual variability has failed. A single measure has little value as an indicator, but correlations between symptoms and fluctuation in FeNO values add relevant data of asthma severity and control.[59] Changes in FeNO prior to moderate exacerbations have been observed.[60]

Konradsen et al. [61] detected in both exhaled FeNO and blood eosinophils a high predictive value for the recognition of those patients with less asthma control. However, it is important to highlight that this accuracy decreases when children are under asthma medication.[55]

Petsky et al. [62] assessed whether an asthma management based on FeNO levels, reduces asthma exacerbations compared with treatment based only in symptoms reported. On the other hand, Pijnenburg et al. [63] did not observe an improvement of airway hyperresponsiveness and inflammation after 1 year of steroid titration on FeNO. Although the addition of FeNO to standard strategy of asthma care saves more than 600 US dollars per patient per year comparing to costs due to asthma exacerbation,[64] whether this management is beneficial for improving asthma control in a short term is still unclear.[62] One of the reasons may be that FeNO levels are more related to atopy than to asthma control.[65]

Nevertheless, treatment decisions based on FeNO may lead to relevant long-term asthma management implications.[64]

Duijts et al. [66] reported higher levels of FeNO in adolescent patients who were early-onset wheezers and worse asthma control throughout childhood.

As observed in younger children, higher FeNO indicates worse asthma severity in a population of inner-city adolescents.[67] Karlin et al. [68] found elevated FeNO during asthma exacerbations, which decreased when asthma control improved.

The recommendations in daily clinical practice are that, as biomarker of Th2 inflammation, FeNO may be useful as predictor of asthma, but with limited value for asthma diagnosis and evaluation of adherence to inhaled corticosteroids.

## Other biomarkers

### Periostin

There are limited studies that support periostin usefulness in asthmatic children. Published works that examine the relationship of this protein with severity or inflammatory pattern of asthma in children are very scarce, with little conclusive data.[69]

Periostin levels are known to be higher in children than in adults, as a result of the cell turnover that occurs during growing period.[70] It can be elevated in intercurrent processes such as rhinosinusitis, polyposis or atopic dermatitis.

Lopez-Guisa et al. [71] observed that in asthmatic children, periostin was 3.7-fold higher in bronchial cells (*p* < 0.001) and 3.9-fold higher in nasal cells (*p* < 0.04) compared to periostin in atopic, non-asthmatic or healthy children. Song et al. found significantly higher periostin values in children with asthma compared with healthy children (76 [65–91.8] vs. 71 [57.5–80] ng ml^–1^), and a relationship between levels of serum periostin and response to mannitol and methacholine.[72] These authors detected significant correlation between serum periostin, blood eosinophil count and FeNO. Moreover, Konradsen and his working group [61] analyzed biomarkers of Th2-inflammation in 96 children with persistent asthma, finding levels of eosinophils and FeNO to have a high predictive value for identifying severe asthma (SA), and found no relationship between periostin and SA. Recently, Inoue et al. [73] suggested that measuring levels of serum periostin combined with the measurement of FeNO, eosinophilia and lung function could improve the diagnosis of asthma in children. They studied 28 children with controlled persistent asthma and 27 healthy children (6–16 years) and assessed utility of serum periostin levels in diagnosing pediatric asthma, as they detected periostin values equivalent to conventional biomarkers, confirming the utility of serum periostin levels in diagnosing asthma in children.[73] Habernau et al. [74] measured serum periostin in a group of children (5–14 years old) with uncontrolled asthma and without correct treatment, and they found higher periostin levels (average 900 ng dl^–1^) compared to previous studies published in children with controlled asthma and with adequate treatment following clinical guidelines.

It seems that periostin is involved in inflammatory process of asthma, with levels decreasing with corticosteroids and it could be useful for monitoring disease progression. Further studies are needed on the role of periostin as an asthma biomarker in childhood asthma.

### Biomarkers in exhaled breath condensate

Exhaled breath condensate (EBC) is a non-invasive local biomarker source to measure the exhaled air non-volatile compounds.

Elevated levels of hydrogen peroxide, nitrates and nitrites [75] and leukotrienes (LT) B4 [76] have been found in the EBC in children with asthma. Asthma control has been related to the IFN-gamma and the IL-4 and IL-5.[77] Exhaled leukotrienes levels are negatively related with BHR.[78] Higher levels of 8-isoprostano and LT were detected both in exercise-induced asthma [79] and severe asthma.[80]

EBC-pH values in asthmatic children are lower than in healthy children.[81] Decrease of pH in EBC has been related to airway inflammation. Caffarelli et al. [82] found a relationship between lower exhaled pH levels in children with asthma exacerbation before treatment, but with no differences between asthmatic children after treatment and healthy children. As conclusion, pH value in EBC is an indicator of acute exacerbation in asthmatic children.[83]

This biomarker could be useful in the diagnosis and follow of asthmatic children in the future.

The main limitation of this technique is the great variability in measurement as it can be modified by environmental pollution or infections. Moreover, EBC is a time-consuming test that still needs methodological validation.

### Volatile organic compounds measured by electronic nose

This is a novel approach to analyze volatile organic compounds (VOCs) in exhaled breath. This is a promising non-invasive biomarker in children with asthma.[84] There is evidence of specificity and reproducibility of soluble biomarkers measurements in nasal lavage, in patients with allergic rhinitis.[85] Some authors found significant differences in VOC in eosinophilic and neutrophilic asthmatics. Therefore, an electronic nose would be a simple, easy to use, and novel technology with clinical value for the inflammatory phenotyping of asthma.[86]

### Thymic stromal lymphopoietin

Thymic stromal lymphopoietin (TSLP) is an epithelial-derived cytokine and has a relationship with Th2 response in asthma. TSLP impairs IL-10 production of Treg cells and inhibited their suppressive activity. Increased levels in asthmatic children were detected. This protein has been related with asthma control and may be used as a biomarker for inflammation in childhood asthma,[87] although there are no differences between allergic and non-allergic childhood asthma.[88]

### Urinary leukotrienes

Leukotriene metabolite count in urine (U-LT) is a non-standardized test related to the measurement of total LT production. Cysteinyl leukotrienes C4 (LTC4) and D4 (LTD4) are lipid mediators that play an important role in the pathogenesis of asthma. They can be secreted by several cells (e.g. eosinophils, neutrophils, and mast cells). LTC4 and LTD4 are rapidly converted to a less active metabolite LTE4, which will be partly eliminated in the urine.[89]

There are higher levels of U-LT inacute wheeze episode in preschool atopic children, compared to non-atopic.[90] The measurement of U-LT could be a non-invasive method for atopic predisposition and diagnosis and management of asthma in preschool children.[91] It is known that environmental tobacco smoke exposure is associated with higher U-LT.[92]

### Genetic biomarkers

There are numerous studies showing that asthma has genetic components. These include the locus ORMDL3, ADAM33, and several cytokine or cytokine receptor genes (IL-18R1, IL-333, IL-2RB, IL-10, TGFB, and IL-6R).[93] The genes for IL-33 and TSLP have emerged as two of the most important associations for the development of the asthma.[87] Pharmacogenomics, play an important role in the efficacy and concentration of the drug. Variations have already been found in STIP1, responsible for glucocorticoid receptor. Polymorphism in the FCER2 gene, which encodes the low affinity IgE receptor, is related to an increased risk of hospital visits for asthma attacks and high doses of daily oral corticosteroids.[94]

In children, a better response to inhaled corticosteroids (ICS) has been associated with variations of TBX21, and asthmatic children homozygous for the variant of the beta-adrenergic receptor gene had little response when a long-acting beta2 agonist (LABA) was added to treatment with ICS.[95] All of these studies are limited by the small sample and heterogeneous population. Specifically, genetic approaches have not shown clinical utility to date.

## Summary

Pediatric asthma is an enormous challenge for clinicians, not only for diagnosis but also in terms of prognosis and follow-up.[96] An inflammatory biomarker, giving additional information, would represent an immense benefit in daily clinical practice. However, at present, an optimal inflammometry biomarker does not exist.

The measurement of the Th2 inflammation in pediatric asthma is achieved with serum tIgE, blood eosinophilia and FeNO levels that may help to predict asthma, particularly in infants and young children in whom lung function tests are difficult to perform.[47] Therefore, a combination of atopy biomarkers, such as sensitization to allergens measured by SPT and/or sIgE, with tIgE, FeNO and blood eosinophilia are related with risk of asthma development in school age.[18,20,21] They can be useful also as markers of severity in childhood asthma.[23,40,57] Intra-individual levels of FeNO may also be useful for ascertaining treatment adherence. Nevertheless, limited or insufficient data were found on biomarkers for pediatric asthma diagnosis, according to literature reviewed (Table 1).

**Table 1.** Routine inflammometry biomarkers of pediatric asthma and their utility in clinical practice*.

	Predictor of asthma development	Diagnosis of asthma	Adherence to treatment	Follow-up	Severity
tIgE	Limited	Limited	Inappropriate	Limited	Limited
Blood eos	Applicable	Inappropriate	Not enough data	Limited	Limited
SPT	Applicable	Not enough data	Not enough data	Applicable+	Applicable
sIgE	Applicable	Not enough data	Not enough data	Applicable+	Applicable
FeNO	Applicable	Limited	Limited	Controversial	Controversial

*According to indexed references reviewed

+Sensitization to certain perennial aeroallergens are indicators of severity/persistence of symptoms.

***Applicable***: Data suggest strongly that this biomarker is useful for the clinical issue indicated.

***Limited***: Data reviewed are scarce about the utility of this biomarker for the clinical issue indicated, but weakly suggest that this biomarker is useful for the clinical issue indicated.

***Not enough data***: Data reviewed are scarce about the utility of this biomarker for the clinical issue indicated, with no conclusions obtained.

***Controversial***: Data reviewed suggest both the utility and inappropriateness of the biomarker for the clinical issue indicated.

***Inappropriate***: Data suggest strongly that this biomarker is not useful for the clinical issue indicated.

Elevated blood eosinophil and aeroallergen sensitization is associated with the increased risk of asthma in children.[18,20,39]

An isolated measurement may be insufficient and only the combination of these markers, along with medical history and lung function, improves the diagnosis, phenotyping and follow-up of an asthmatic child. Publications about pediatric asthma show controversial results. To date, there is no definitive biomarker to identify children with high-risk phenotypes that will develop severe persistent asthma.

Prior to determining which is the perfect biomarker for pediatric asthma, clinicians should determine the characteristics of the population studied and the characteristics of the asthma disease they would like to measure.
